# Characterization of a *Lactobacillus gasseri* strain as a probiotic for female vaginitis

**DOI:** 10.1038/s41598-024-65550-y

**Published:** 2024-06-23

**Authors:** Jingyan Zhang, Kailing Li, Tishuang Cao, Zhi Duan

**Affiliations:** Qingdao Vland Biotech Group Co., Ltd, Qingdao, China

**Keywords:** Biotechnology, Microbiology

## Abstract

Vaginitis, a prevalent gynecological condition in women, is mainly caused by an imbalance in the vaginal micro-ecology. The two most common types of vaginitis are vaginal bacteriosis and vulvovaginal candidiasis, triggered by the virulent *Gardnerella vaginalis* and *Candida albicans*, respectively. In this study, a strain capable of inhibiting *G. vaginalis* and *C. albicans* was screened from vaginal secretions and identified as *Lactobacillus gasseri* based on 16S rRNA sequences. The strain, named *L. gasseri* VHProbi E09, could inhibit the growth of *G. vaginalis* and *C. albicans* under co-culture conditions by 99.07% ± 0.26% and 99.95% ± 0.01%, respectively. In addition, it could significantly inhibit the adhesion of these pathogens to vaginal epithelial cells. The strain further showed the ability to inhibit the enteropathogenic bacteria *Escherichia coli* and *Salmonella enteritidis*, to tolerate artificial gastric and intestinal fluids and to adhere to intestinal Caco-2 cells. These results suggest that *L. gasseri* VHProbi E09 holds promise for clinical trials and animal studies whether administered orally or directly into the vagina. Whole-genome analysis also revealed a genome consisting of 1752 genes for *L. gasseri* VHProbi E09, with subsequent analyses identifying seven genes related to adhesion and three genes related to bacteriocins. These adhesion- and bacteriocin-related genes provide a theoretical basis for understanding the mechanism of bacterial inhibition of the strain. The research conducted in this study suggests that *L. gasseri* VHProbi E09 may be considered as a potential probiotic, and further research can delve deeper into its efficacy as an agent which can restore a healthy vaginal ecosystem.

## Introduction

The female vagina is a dynamic microecological environment, where balance is largely maintained by the vaginal microflora which is dominated by *Lactobacillus* species^1^. Indeed, in women of childbearing age, the presence of this particular genus has long been recognized as essential for a healthy vaginal environment^1^. So far, approximately 25 different types of lactobacilli have been reported as forming a complex population in the vagina, and these include *Lactobacillus crispatus*, *Lactobacillus gasseri*, *Lactobacillus iners* and *Lactobacillus jensenii* as the predominant species^2–6^. Given their importance, any imbalance in the vaginal microbiota that reduce *Lactobacillus* levels can subsequently allow the emergence of other dominant endogenous or exogenous bacteria. This can result in a number of gynecological disorders that get translated into physical and mental discomfort which eventually affect a woman’s daily life. Some of the outcomes of such an imbalance in the vaginal microecological environment include vaginal bacteriosis, cytolytic vaginosis, vulvovaginal candidiasis, trichomonas vaginitis, urinary tract infection and other infectious diseases of the female genitourinary tract^7–9^.

Bacterial vaginitis (BV) is characterized by elevated vaginal pH, malodorous discharge, and it is considered to be a polymicrobial condition in which pathogenic bacteria, predominantly *Gardnerella vaginalis*, form a biofilm on the vaginal wall^10–13^. Vulvovaginal candidiasis (VVC), was reported to be the second most common form of vaginitis, with 75% of female patients being women of childbearing age^14,15^. In this case, 90% of VVC infection is primarily due to the attachment of *Candida albicans* to vaginal cells to form a biofilm^15–17^.

Currently, the main clinical strategy for treating BV involves the use of antibiotics such as metronidazole, clindamycin and tinidazole^18^, with short-term cure rates close to 80%^19^. VVC is primarily treated with antifungal drugs, such as azols and ibrexafungerp^20^. It has also been reported in the literature that topical boric acid and flucytosine can also be therapeutic^21^. However, vaginitis often recurs after drug treatment, and this highlights the need to identify safe and effective alternative treatments to alleviate the physical and psychological burden on patients.

*Lactobacillus* can maintain the ecological balance of the genitourinary tract through various mechanisms such as host immune regulation, recovery of the vaginal microbiota and interference with pathogen colonization^22,23^. In addition, lactobacilli can also inhibit the growth of pathogenic bacteria through the production of bacteriostatic substances such as hydrogen peroxide and bacteriocins, while being able to competitively repel such pathogens through the production of adhesins^24–26^. Clinical studies have shown that orally or vaginally administered microecological agents can significantly reduce morbidity and recurrence of vaginitis^27–29^. For instance, Shamshu et al.^30^ noted the therapeutic effects of *Lactobacillus rhamnosus* GR-1 and *Lactobacillus reuteri* RC-14 on reproductive tract infections, while Reznichenko et al.^31^ reported reduced BV recurrence and prolonged duration of recurrence with *Lactobacillus crispatus* LMG S-29995, *Lactobacillus brevis* Lbr-35 and *Lactobacillus acidophilus* La-14. Similarly, Rosario Russo et al.^32^ showed that a combination of *Lactobacillus acidophilus* GLA-14 and *Lactobacillus rhamnosus* HN001 with lactoferrin reduced the symptoms and recurrence of VVC.

The objectives of this study were to isolate *Lactobacillus* strains from vaginal secretions before identifying the isolates and assessing their safety. The in vitro probiotic effects of the isolates against *Gardnerella vaginalis* and *Candida albicans* were then determined. It is expected that the results of this study will assist the introduction of new probiotics with therapeutic potential for promoting women’s health.

## Materials and methods

### Isolation and identification of LAB strains

According to the 2019 “Implementation Rules of Administrative Regulations on Human Genetic Resources”, samples were obtained in accordance with the biobank’s standard operating practices after securing informed consent from the sample provider^33^. The general procedure is to sign an informed consent form with the volunteers who participate in the sampling, then provide the volunteers with the sampling tools and sampling procedure, and the volunteers take the samples by themselves and deliver the samples to the laboratory. Lactic acid bacteria (LAB) were then isolated from these samples using MRS medium. Both *Gardnerella vaginalis* and *Candida albicans* were commercially purchased strains (BeNa, China).

Taxonomic classification of the isolates was achieved through 16S rRNA sequence analysis by following methods reported in literature^34^. New isolates were also identified by database sequence comparison. In addition, sequences of closely related strains were retrieved from the GenBank Library Project database to construct a phylogenetic tree, based on the Neighbor-Joining method, using MEGA 11.0 software.

### Antimicrobial activity

The Oxford Cup method was used to evaluate the inhibitory activity of the isolated strains against *G. vaginalis*^35,36^. For this purpose, 50 µL of *G. vaginalis*, at a concentration of about 10^9^ CFU/mL, was evenly spread on Columbia Blood Agar plates using a sterile applicator stick before adding 100 µL of bacterial test strain solution (10^9^ CFU/mL) to determine its bacteriostatic ability. The inhibitory effects of the isolates against *C. albicans* were then tested using the method of Zhang et al.^37^. 5 µL of the isolate broth (10^9^ CFU/mL) was dripped onto the prepared MRS agar plate, then dried and cultured for 48 h to allow the colony to grow. Then 5 mL of semi-solid Sabouraud Dextrose Agar (SDA) medium containing *Candida albicans* (10^6^ CFU/mL) was poured onto the plate containing the colony, dried and cultured for 24–48 h. In both cases, the diameter of inhibition zones, indicative of the inhibitory activity of the isolates, was evaluated.

The enteropathogenic bacteria *Escherichia coli* and *Salmonella enteritidis* also served as indicator strains for testing the bacteriostatic capacity of the isolates using the Oxford cup method. All experiments were conducted in triplicate.

### Hydrogen peroxide production

H_2_0_2_ production by LAB was determined using the method of McGroarty et al.^38^. Briefly, isolates on MRS plates containing 10 g/L glucose, 0.25 g/L tetramethylbenzidine (TMB, Macklin, China) and 0.01 g/L horseradish peroxidase (Macklin, China), were incubated anaerobically at 37 °C for 48–72 h. They were then exposed to ambient air, and since in the presence of H_2_O_2_, horseradish peroxidase in the medium oxidizes TMB to form blue pigments, a color change was indicative to H_2_O_2_,-producing colonies.

### Co-culture of LAB strains and pathogens

For this experiment, *G. vaginalis* was cultured in BHI broth medium supplemented with 5% bovine serum, while LAB and *C. albicans* were cultured in MRS Broth and Sabouraud Dextrose Broth, respectively.

Co-culture experiments with LAB strains and pathogens were performed according to the method of Bo Ram Beck er al. to determine bacteriostatic effects^39^. To test activity against *G. vaginalis*, the pathogen and LAB (both at 10^9^ CFU/mL) were inoculated at 1% (v/v) inoculum into BHI broth containing 5% bovine serum prior to aerobic incubation at 37 °C for 24 h. Similarly, to determine activity against *C. albicans*, MRS broth (10 mL) was inoculated with 1.0% (v/v) of LAB (10^9^ CFU/mL) and 1.0% (v/v) of *C. albicans* (10^7^ CFU/mL). This was followed by a 24 h aerobic incubation at 37 °C. Media inoculated with *G. vaginalis* or *C. albicans* alone were used as the control. The viable bacteria count (CFU/mL) of each pathogen in the experimental and control groups were then measured. The selective media used were Sabouraud Dextrose Agar (SDA) medium for *C. albicans* and Columbia Blood Agar (CBA) medium for *G. vaginalis*. The inhibition rate (%) was eventually calculated as follows:

$${\text{Inhibition}}\;{\text{ rate }}\left( \% \right) \, = \, \left( {{1}{-}{\text{Count }}\;{\text{in }}\;{\text{pathogen}}\;{\text{ and}}\;{\text{ LAB}}\;{\text{ mixture}}/{\text{Count}}\;{\text{ in}}\;{\text{ pathogen}}\;{\text{ control}}} \right) \, \times {1}00$$

### Adhesion test on Caco-2 and vaginal epithelial cells

Caco-2 and vaginal epithelial cells were purchased from Shanghai Goyan Bio. Co. (Shanghai, China). The adhesion experiment was then carried out according to previous research, with some modifications^40^.

Vaginal epithelial cells were cultured in specialized medium (Goyan Bio., Shanghai), while Caco-2 cells were cultured in RPMI-1640 medium containing 10% (v/v) fetal bovine serum and 1% (v/v) penicillin–streptomycin solution. The adhesion test was then performed in a 24-well plate, and briefly, this involved adding 2.5 × 10^5^ cells to each well prior to a 24-h incubation at 37 °C to allow cell attachment. The number of cells was calculated using a blood cell counting board. The medium was subsequently discarded and after washing the wells twice with phosphate buffer solution (PBS, pH 7.0) to remove unattached cells, 1 mL (10^7^ CFU/mL) of bacterial suspension was added to the wells. This was followed by a 2-h incubation at 37 °C and under 5% CO_2_ to allow bacterial adhesion to the cells. Unstuck bacteria were then removed by adding 300 uL of pancreatin to detach the cells from the wall before adding 700 uL of culture solution to stop digestion. The number of viable bacteria was finally measured by plate counting method, with the adhesion index calculated using the following formula:

$${\text{Adhesion}}\;{\text{ index }} = {\text{ number}}\;{\text{ of}}\;{\text{ adhered }}\;{\text{bacteria }}/{\text{number}}\;{\text{ of}}\;{\text{ cells}}.$$

### Inhibition of pathogen adhesion to vaginal epithelium

The vaginal epithelial cell adhesion assay was used to evaluate the inhibitory effects of LAB against *G. vaginalis* and *C. albicans*^25,37^. In this assay, the concentrations of both LAB and pathogenic bacteria were adjusted to 1.0 × 10^7^ CFU/mL, with 2.5 × 10^5^ cells subsequently added to each well of a 24-well plate. A mixture of LAB and pathogen suspensions (500 μL), prepared from equal volumes of each, was then added to the plate. In this experiment, vaginal epithelial cells incubated with *G. vaginalis* or *C. albicans* alone acted as the controls. All tests were conducted in triplicate, and the number of pathogenic bacteria in the experimental and control groups was determined separately. The pathogen adhesion indexes of the control group were finally compared with those of the experimental group, with the difference reflecting the test strain’s inhibitory effects on pathogen adhesion.

### Tolerance to simulated gastrointestinal juice

The survival rate of LAB in the gastrointestinal (GI) system was assessed using the method of Millette et al.^41^ with slight modifications. Firstly, the pH of simulated gastric juice was adjusted to 3.0, while that of simulated intestinal fluid (SIF) was adjusted to 6.8 before autoclaving. Prior to testing, the bacteria were also washed three times with an equal volume of phosphate buffer saline (PBS, pH 7.0). The bacterial concentration was then adjusted to 10^9^ CFU/mL, and after adding 1 mL of the bacterial suspension to 9 mL of simulated gastric fluid, incubation was performed for 2 h at 37 °C with continuous shaking at 200 rpm. This was followed by the transfer of 1 mL of the resulting mixture to 24 mL of SIF, and after a second incubation (3 h at 37 °C with shaking at 200 rpm), the gastrointestinal tolerance of LAB was assessed by comparing bacterial counts before and after gastrointestinal transit.

### Whole genome sequencing and analysis

Bacteria cultured for 22 h were collected by centrifugation at 10,000×*g* for 10 min, and after being quickly frozen in liquid nitrogen, they were sent to Majorbio Sequencing Center (Shanghai, China) on dry ice for whole genome sequencing and analysis. The genome was sequenced using a combination of the Illumina Hiseq 2500 and PacBio RS II single-molecule real-time (SMRT) sequencing platforms^42^. The number of genes, gene functions, virulence factors and repressor genes were then analyzed using available software before performing gene function annotations with the NR and KEGG databases. Antimicrobial resistance genes were analyzed with the ResFinder software. In this case, gene function was compared using BLAST + software.

### Statistical analysis

All tests were performed in triplicate, with the differences between treatments analyzed using 2-tailed Student’s t-tests in Excel software (Microsoft, Redmond, WA, USA). When *p* ≥ 0.05, the difference is not significant; when *p* < 0.05, the difference is significant; and when *p* < 0.01, the difference is extremely significant.

### Ethical approval

All methods were carried out in accordance with relevant guidelines and regulations.

## Results

### Screening of bacterial isolates and identification

A total of eight *Lactobacillus* strains, screened from vaginal secretions, were able to inhibit *G. vaginalis* to varying degrees, with the size of the inhibitory zones produced by each strain shown in Table 1. Specifically, except for E08 and E11, the remaining six strains had nearly similar inhibitory potential. Through additional screening involving *Candida albicans* inhibition, it was found that only strain E09 could inhibit the second pathogen with inhibitory diameters of 1.30 ± 0.1 cm on agar plates. Therefore, E09 was selected as a candidate probiotic strain for subsequent experiments. Interestingly, the selected strain was also able to inhibit the growth of *E. coli* and *S. enteritidis* with inhibitory diameters of 1.13 ± 0.06 cm and 1.44 ± 0.02 cm, respectively. Finally, additional tests revealed that strain E09 could produce hydrogen peroxide, with its colonies turning blue on media containing horseradish peroxidase.

**Table1:** Antibacterial effects of isolated LAB on *G. vaginalis* as indicated by the size of their zones of inhibition (in cm).

LAB	Zone of inhibition, cm
E01	1.47 ± 0.15
E04	1.40 ± 0.00
E07	1.30 ± 0.00
E08	1.03 ± 0.06
E09	1.40 ± 0.00
E10	1.33 ± 0.12
E11	1.13 ± 0.12
E12	1.23 ± 0.06

**Figure 1:**
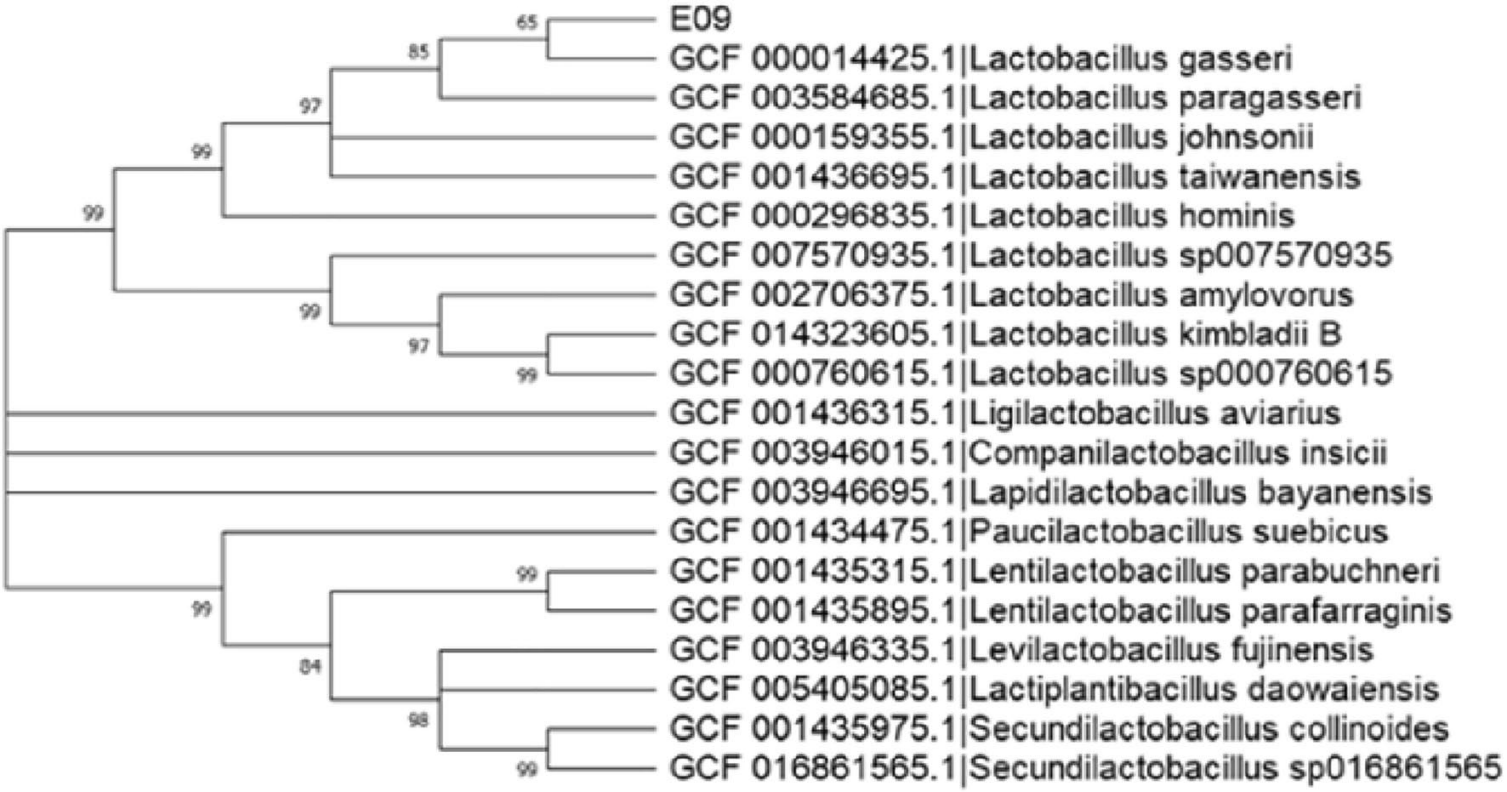
Phylogenetic tree of *L. gasseri* VHProbi E09 based on 16S rRNA sequences.

The 16S rRNA sequence of the strain was uploaded to the NCBI database (Genbank accession: OR945710), and based on BLAST comparison, it was found to be closely related to *L. gasseri*. Nineteen closely related strains were also selected, and their downloaded 16s rRNA sequences were used to construct a phylogenetic tree, based on the Neighbor-Joining method, using MEGA11.0 software (Fig. 1). Overall, strain E09 showed the highest homology to *L. gasseri* GCF 000,014,425, and hence, it was named *L. gasseri* VHProbi E09.

### Inhibitory effects of *L. gasseri* VHProbi E09 against pathogens

**Figure 2:**
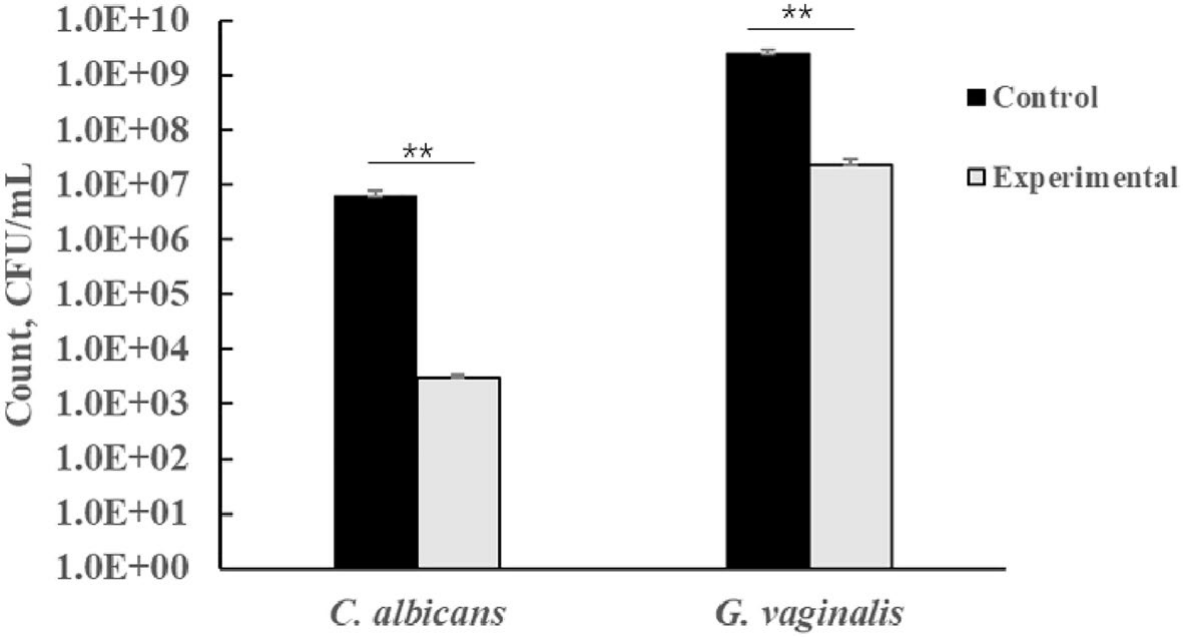
The inhibitory effects of *L. gasseri* VHProbi E09 against *C. albicans* and *G. vaginalis* by co-culture method. ∗ *p* < 0.05, ∗  ∗ *p* < 0.01.

Figure 2 shows the inhibitory effects of *L. gasseri* VHProbi E09 on the growth of *G. vaginali*s and *C. albicans*. For the *G. vaginalis* pathogen, after 24 h of incubation with *L. gasseri* VHProbi E09, the final bacterial load of 2.27 × 10^7^ CFU/mL. Thus, compared with the control which had a bacterial load of approximately 2.43 × 10^9^ CFU/mL, the results represented a 99.07% ± 0.26% inhibition of *G. vaginalis*. Regarding *C. albicans*, the control group had a bacterial load of approximately 6.17 × 10^6^ CFU/mL, while the final bacterial load after adding *L. gasseri* VHProbi E09 was 2.97 × 10^3^ CFU/mL, hence indicating an inhibition of 99.95% ± 0.01%.

### Test of adhesion to vaginal epithelial cells

Adhesion is an important prerequisite for the colonization of probiotics, and in this set of experiments, *L. gasseri* VHProbi E09 was found to adhere strongly to primary vaginal epithelial cells. Specifically, after 2 h of incubation, 1.7 × 10^5^ bacterial CFU could be detected inside each well containing vaginal epithelial cells, with this value indicating an adhesion index of 6.9 ± 1.0 CFU/cell. The adhesion ability of these bacteria highlights their potential to stay in the vagina for a long period of time during which they can exert effective probiotic effects.

*L. gasseri* VHProbi E09 also adhered strongly to Caco-2 cells. In this case, 6.07 ± 10^5^ bacterial cells could be detected inside each well containing Caco-2 cells after 2 h of incubation. Thus, with an adhesion index of 2.43 ± 0.27 CFU/cell, the results suggested that these bacteria could remain in the gut for a long period of time, thereby making them effective in providing extended probiotic effects. In addition, the ability of intestinal *Lactobacillus rhamnosus* GG strain to adhere to Caco-2 cells was determined, but its adhesion index of 1.76 ± 0.22 CFU/cell suggested that it was less effective than *L. gasseri* VHProbi E09.

### Inhibition of pathogen adhesion to vaginal epithelial cells

**Figure 3:**
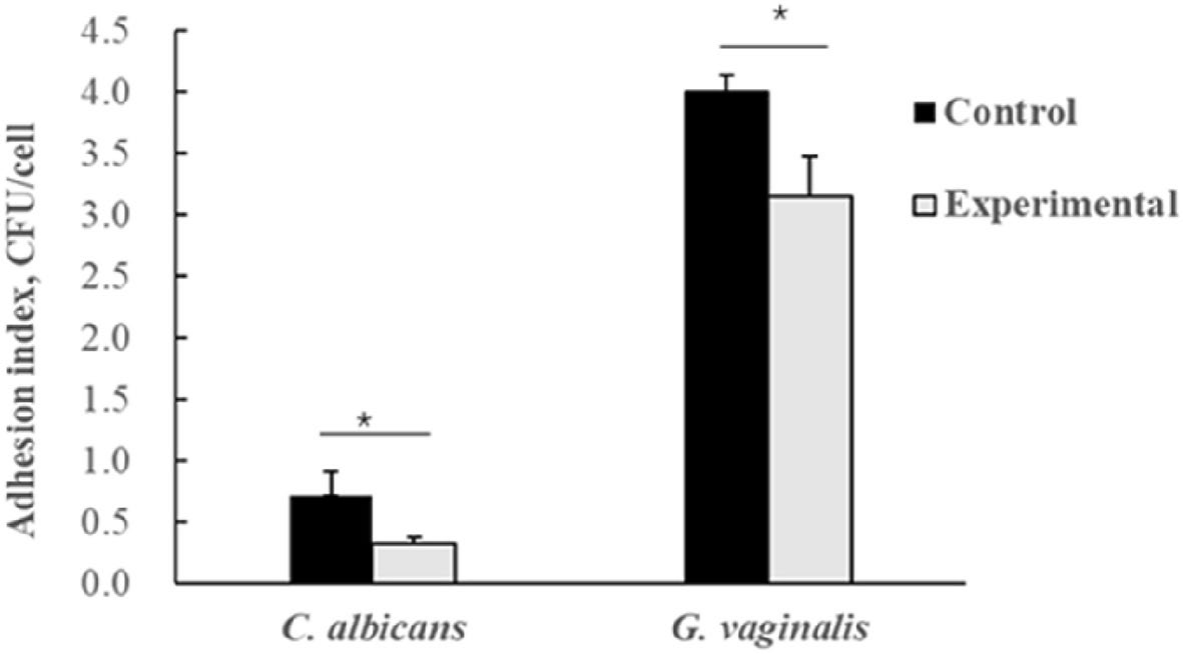
Effects of *L. gasseri* VHProbi E09 on adhesion of *C. albicans* and *G. vaginalis* to vaginal epithelial cells.  ∗ indicates statistical significance at *p* < 0.05.

The ability of *L. gasseri* VHProbi E09 to reduce adhesion of *C. albicans* and *G. vaginalis* to vaginal epithelial cells is shown in Fig. 3. Overall, *L. gasseri* VHProbi E09 significantly inhibited *G. vaginalis*’s attachment to the epithelial cells (Fig. 3), with its adhesion index of 0.33 ± 0.05 CFU/cell being significantly lower than that of the control (0.71 ± 0.20 CFU/cell) (*p* < 0.05). Similarly, the inhibitory effect of *L. gasseri* VHProbi E09 against *C. albicans* adhesion, was also evident, and in this case, the adhesion index was 3.15 ± 0.33 CFU/cell, while that of the control was 4.00 ± 0.14 CFU/cell (*p* < 0.05).

### Tolerance to artificial GI juice

Table 2 shows the bacterial count before and after digestion with the GI fluids (artificial gastric fluid and artificial intestinal fluid). The results showed that *L. gasseri* VHProbi E09 had a higher survival rate in the simulated gastric and intestinal fluids as its initial inoculum decreased from 8.20 ± 0.01 log CFU/mL to a final count of 7.56 ± 0.02 log CFU/mL (*p* < 0.01). This decrease of 0.66 log CFU/mL was lower compared to that of *L. rhamnosus* GG (LGG) for which the bacterial load was reduced by 2.86 log CFU/mL after digestion with the artificial intestinal solutions. Thus, the results suggested that strain E09 had a higher tolerance to the digestive solutions than *L. rhamnosus* GG.

**Table 2:** Bacterial count before and after digestion with artificial gastrointestinal solution (log CFU/mL).

Strains	Initial count	After 2 h in simulated gastric juice	After 3 h in simulated intestinal fluid
E09	8.20 ± 0.01	8.22 ± 0.03	7.56 ± 0.02
LGG	8.94 ± 0.08	8.17 ± 0.03	5.31 ± 0.03

### Whole genome analyses

The genome of *L. gasseri* VHProbi E09 was sequenced on the PacBio SMRT platform, and the whole genome sequence, with a 99.46% coverage, was then uploaded to NCBI database under GenBank and SRA accession numbers CP129028 and SRR27126921, respectively. The results revealed that *L. gasseri* VHProbi E09 had a circular chromosome of 1 864 621 bases and a GC content of 35.23%. In addition, 93 RNA genes and 1752 open reading frames (ORFs) were identified. Specifically, the latter, with an average length of 952.88 bp and a gene density of 0.94, accounted for 89.53% of the whole genome.

**Figure 4:**
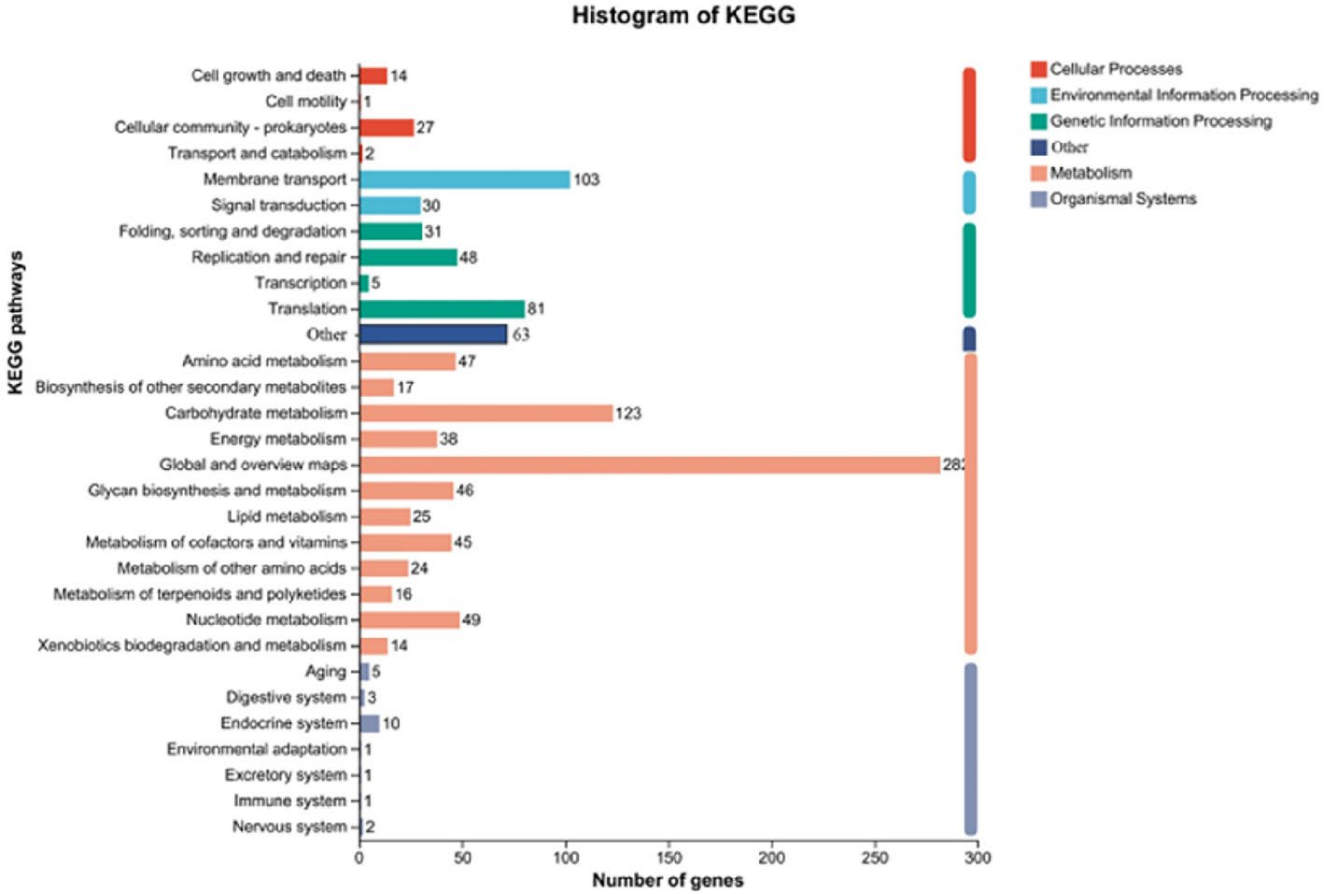
KEGG-based annotation showing the pathways in which *L. gasseri* VHProbi E09 genes could be involved.

Analysis with the ResFinder software (https://cge.cbs.dtu.dk/services/ResFinder/) highlighted the absence of genes associated with antimicrobial resistance in *L. gasseri* VHProbi E09, while the Diamond comparison software further suggested that the isolate had no virulence factor secretion system and hence, may not secrete virulence factors. Annotation of coding genes against the KEGG database subsequently identified 1154 genes which accounted for 65.87% of the total genes (Fig. 4). In particular, 726 genes were involved in metabolism, 165 were involved in genetic information processing, 156 were associated with organismal systems, 133 were involved in environmental information processing, 44 were associated with cellular processes and 63 were involved in other processes. The coding genes were also compared against the NR database, and in this case, seven genes were found to be associated with adhesion. Of these, genes 0044, 0145, 0408 and 0880 were presumed to be associated with adhesion exoproteins, while genes 0878, 0882 and 0883 were presumed to encode adhesins. In addition, there were three genes related to bacteriocin, with gene 0474 which shared 100% similarity to the bacteriocin gene of *Lactobacillus gasseri* (Accession: WP_003647676), presumed to be a class III bacteriocin. Finally, genes 0542 and 0561 were presumed to encode bacteriocin immunity protein.

## Discussion

*Lactobacillus* is generally recognized as the predominant bacterial group in the vagina where it maintains a balance in the microflora by secreting metabolites (such as lactic acid, bactericins, and H_2_O_2_) that inhibit the growth and adhesion of other microorganisms^43,44^. Therefore, *Lactobacillus* strains sourced from vaginal secretions are believed to more effectively colonize and contribute to a healthy vaginal environment. In this study, a *Lactobacillus* strain with potential probiotic effects was screened from vaginal secretions and identified as *Lactobacillus gasseri* based on 16s rRNA sequences. This is not surprising as numerous reports already present *L. gasseri* as a major group of vaginal lactobacilli^45,46^. The isolated strain, referred to as *L. gasseri* VHProbi E09, could inhibit the growth of *C. albicans* and *G. vaginalis* under static and dynamic co-culture conditions. In particular, it could produce hydrogen peroxide (H_2_O_2_) which is known to inhibit a range of pathogens including *G. vaginalis*, *C. albicans* and *E. coli*^44,47,48^. In this context, Eschenbach et al. noted that LAB species that produce H_2_O_2_ may enhance the nonspecific antimicrobial defence of the vaginal ecosystem^49^. Furthermore, whole genome analysis revealed that *L. gasseri* VHProbi E09 harbored three genes encoding bacteriocin, hence indicating its ability to metabolize bacteriocin to inhibit the growth of pathogenic bacteria.

Adhesion ability is crucial for lactobacilli to exert a probiotic role, but at the same time, it is a key process for pathogens such as *G. vaginalis*, *C. albicans* and *E. coli* which cause diseases by adhering to epithelial cells to form biofilms^26,50,51^. Therefore, by colonizing vaginal epithelial cells, LAB can impede the pathogens’ adhesion to the cells, thereby inhibiting their growth^52–54^. Genomic analysis and cell adhesion tests performed in this study showed that *L. gasseri* VHProbi E09 had strong adhesion ability to vaginal epithelial cells, and as such, it could inhibit the adhesion of *Gardnerella* and *C. albicans* to cells. In addition, whole genomic analysis predicted the safety of the isolated strain for human use. Hence, the results suggested that *L. gasseri* VHProbi E09 is a potential vaginal probiotic strain.

Given the significant in vitro inhibition of vaginal pathogens by *L. gasseri* VHProbi E09, future studies should focus on their mechanisms in in vivo models. Vaginal probiotics can prevent and treat vaginitis through oral and vaginal administration^55,56^. The adhesion capacity of *L. gasseri* VHProbi E09, along with the bacteriostatic substances it produces, suggest its enhanced effectiveness through direct vaginal action. Since vaginitis can be treated orally, this study also investigated the isolate’s gastrointestinal tolerance, intestinal cell adhesion and ability to inhibit intestinal pathogens. In this case, it was observed that of *L. gasseri* VHProbi E09 could inhibit the intestinal pathogens *E. coli* and *S. enteritidis*. Furthermore, it had better tolerance to gastric and intestinal fluids as well as better adhesion to intestinal Caco-2 cells compared with the well-known intestinal probiotic strain *L. rhamnosus* GG. Therefore, it is hypothesised that the strain could also exert its probiotic effects in the gut through oral administration.

In conclusion, in vitro experiments indicated that *L. gasseri* VHProbi E09, a hydrogen peroxide producer, could inhibit the adhesion of pathogenic bacteria to cells and tolerate gastrointestinal stress, thereby showing promise as a vaginal probiotic. These findings not only provide a theoretical basis for its later clinical studies, but also offer new ideas and opportunities for the treatment of vaginal-associated infections.

## Data Availability

Sequence data that support the findings of this study have been deposited in NCBI database. GenBank for 16s DNA is OR945710. GenBank and SRA accession numbers for the whole genome are CP129028 and SRR27126921. The datasets generated for this study are available on request to the corresponding author.
